# Delayed Repair of Aortic Dissection in a Trauma Patient With Occult Esophageal Rupture

**DOI:** 10.1016/j.atssr.2023.02.021

**Published:** 2023-03-08

**Authors:** Chang Liu, Yi Song, Ye Yuan, Jianming Chen, Yijie Hu

**Affiliations:** 1 Department of Cardiovascular Surgery, Daping Hospital, Army Medical University, Chongqing, China

## Abstract

Aortic dissection and esophageal rupture are life-threatening conditions in trauma patients. The combination complicates treatment. Here, we report a case of traumatic aortic dissection with occult esophageal rupture that was treated successfully with a staged operative strategy: primary esophageal repair followed by delayed aortic arch and aortic valve repair.

Although aortic dissection (AD) occurs in <1% of blunt trauma patients, it represents the second leading cause of death after head injury.^1^ Coexisting esophageal rupture may complicate treatment of aortic injury. Here, we report the successful staged treatment of a patient with traumatic AD combined with occult esophageal rupture.

A 54-year-old man presented to the local emergency department after blunt chest trauma in a traffic accident. On examination, he was hemodynamically stable with significant blood pressure difference between the arms (right arm, 160/35 mm Hg; left arm, 90/40 mm Hg). The diastolic decrescendo murmur was heard at the left sternal border. Laboratory studies showed a high white blood cell count (26.6 × 10^9^/L), reduced hemoglobin level (119 g/L), increased D-dimer level (33450.47 μg/L), and low fibrinogen concentration (0.77g/L). Transthoracic echocardiography revealed severe aortic regurgitation. Computed tomography (CT) showed limited aortic arch dissection between the brachiocephalic artery (BCA) and left common carotid artery (LCCA) and mediastinal hematoma (Figures 1A-1C). Emergent operation was planned.

**Figure 1:**
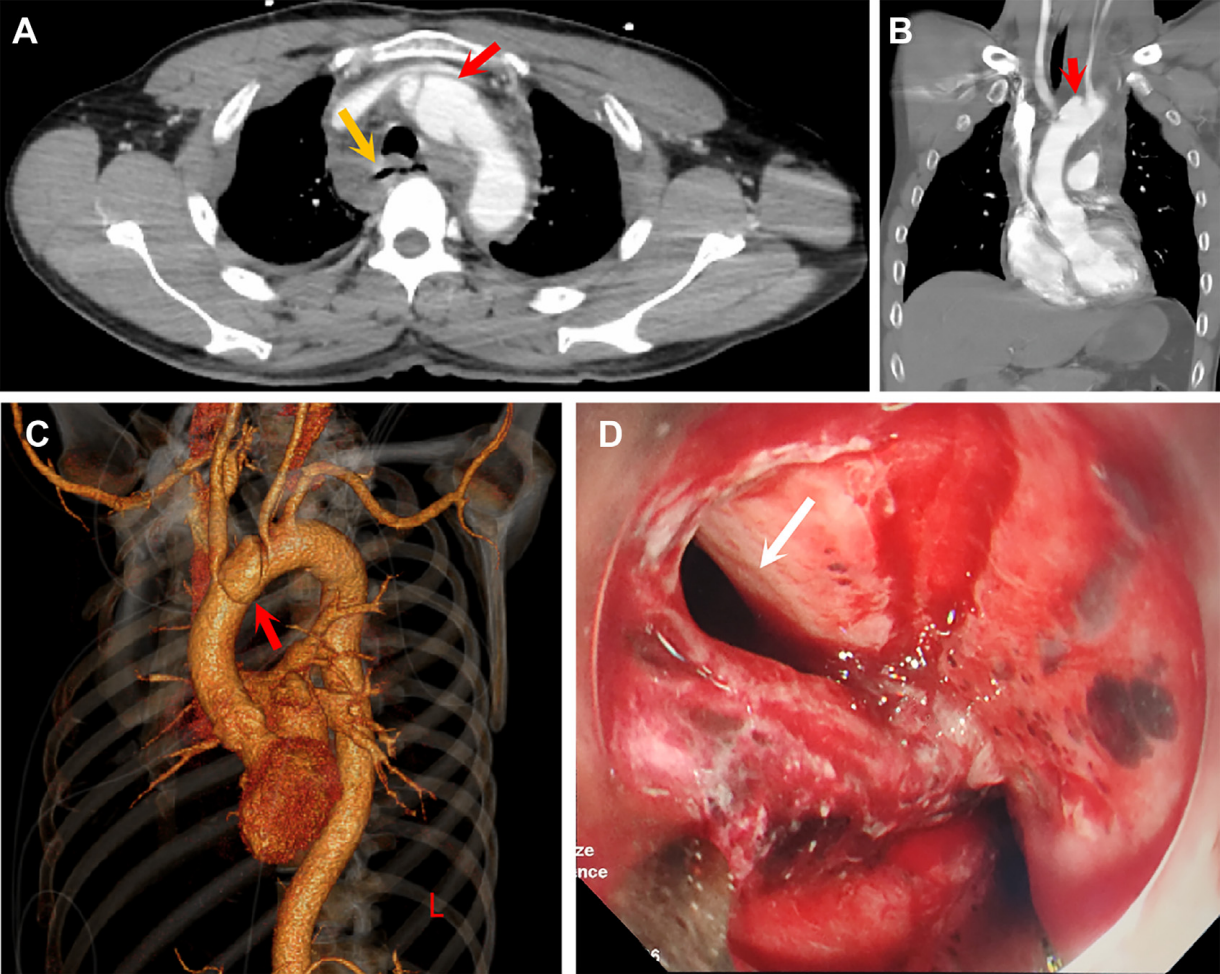
(A-C) Preoperative chest computed tomography showing laceration of the aortic arch (red arrow) and discontinuity of the esophageal lumen (yellow arrow). (D) Esophageal rupture on upper endoscopy (arrow).

However, further analysis of chest CT revealed esophageal wall thickening, gas gathering around the esophagus, and suspicious discontinuity of the lumen at the aortic arch plane (Figure 1A). Upper endoscopy was performed after induction of anesthesia in the operating room. Unexpectedly, a 4-cm-long full-thickness rupture was found on the lateral aspect of the esophagus, spanning 26 to 30 cm apart from the incisors, with extensive superficial ulcer around and active bleeding (Figure 1D).

Considering that there was a low risk of AD progression, whereas esophageal injury might lead to the development of severe mediastinal sepsis and disorder of the coagulation-fibrinolysis system, we decided to repair the esophagus in the primary operation, which was no more than 6 hours from injury. The thoracic esophageal injuries were approached through a right anterolateral thoracotomy in the third intercostal space. The perforated esophagus was repaired directly in 2 layers, with an inner running suture and an outer interrupted suture. On day 5 of admission, a tracheotomy was performed because of extensive pulmonary contusion and respiratory distress. The esophagus healed fully 2 weeks later, confirmed on endoscopy. However, it was difficult to wean the patient from mechanical ventilation owing to anterolateral flail chest. Hence, the fractured ribs were fixed with surgical plating on day 29 of admission. On day 40 of admission, the patient was decannulated. During that period, the patient was hemodynamically stable with no obvious progression of the aortic lesion on serial contrast-enhanced CT assessment.

On day 79 after the traffic accident, the patient accepted operation for aortic arch dissection and severe aortic regurgitation. After median sternotomy, the aortic arch and its branches were circumferentially dissected and controlled with vessel loops. Cardiopulmonary bypass was then initiated with femoral/axillary arterial cannulation and double-stage venous cannulation into the right atrium. Inspecting the aortic root, we found that there were 1 or 2 liner tears or perforation in each aortic leaflet without any prolapse. The perforated leaflets were repaired with 5-0 Prolene interrupted or continuous sutures. Then, using unilateral antegrade cerebral perfusion with occlusion of the BCA, LCCA, and distal aortic arch, we explored the aortic arch through a transverse incision on the proximal part of the aortic arch. Complete circumferential detachment of the intima was noted just between the BCA and LCCA. We used a short segment (10 mm in width) of 26-mm tubular graft to reconnect the 2 detached parts by full-layer continuous suturing with 4-0 HEMO-SEAL Prolene (Figure 2). Postoperative transesophageal echocardiography demonstrated no aortic regurgitation.

**Figure 2:**
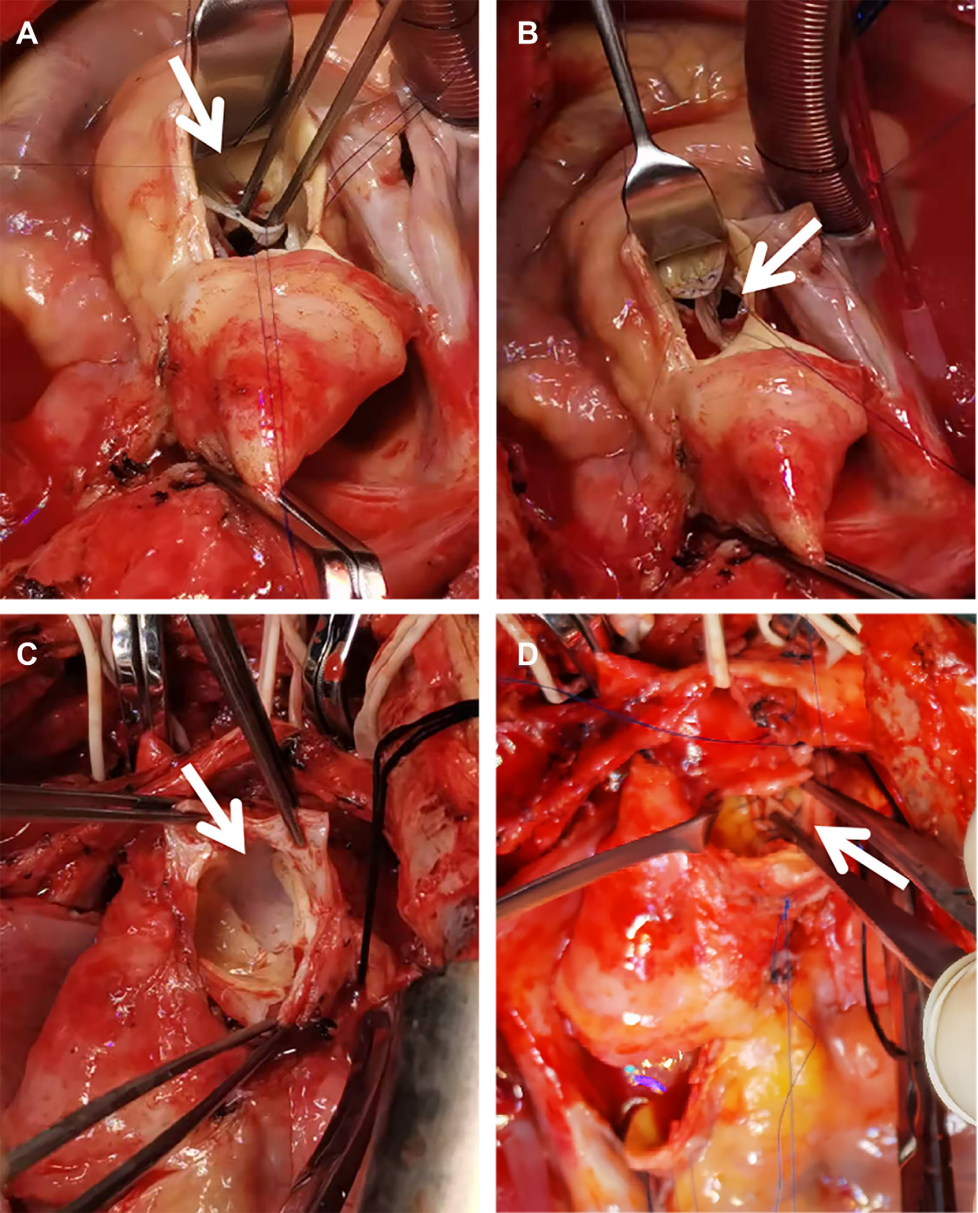
Repair of aortic valve and aortic arch dissection. (A, B) Perforation of coronary leaflet (arrow). (C) Complete circumferential detachment of the intima between brachiocephalic artery and left common carotid artery (arrow). (D) The dissected aorta was repaired with a short segment of tubular graft patch (arrow).

At 6-month follow-up, chest CT showed no residual dissection or leak in the ascending aorta and arch, and chest radiography showed normal contours of the heart and mediastinum (Figure 3). Echocardiography reported mild central aortic regurgitation.

**Figure 3:**
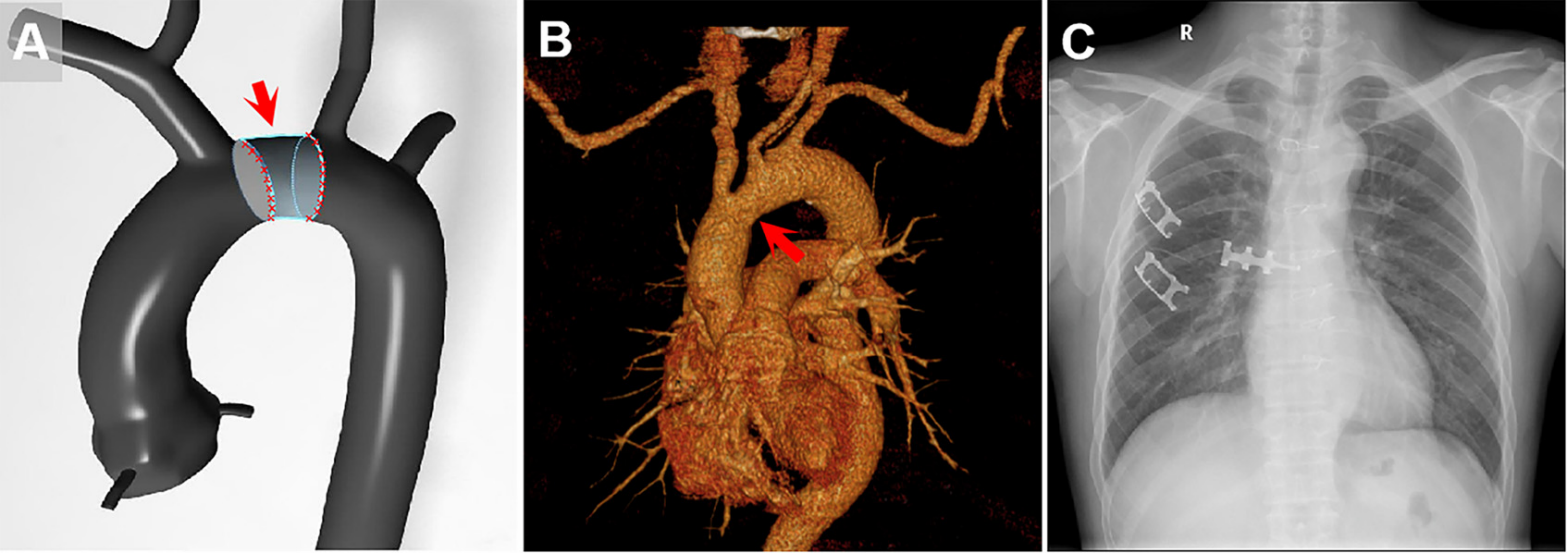
Follow-up at 6 months after repair of aortic dissection. (A) Schematic diagram of limited aortic repair (arrow). (B) Three-dimensional review of repaired aortic arch (arrow). (C) Postoperative chest radiograph.

## Comment

AD and esophageal rupture are life-threatening conditions in trauma patients. The combination may complicate treatment and lead to a poor outcome.^2^ Prioritization of which condition should be approached first must consider the overall risk-benefit equation specific to a particular clinical scenario.

Esophageal injury as a result of blunt trauma is an exceedingly rare event, occurring in <0.1% to 1.5% of patients, and is often a diagnostic challenge, especially because of the potential for damage to surrounding tissues and concurrent contamination. Early diagnosis of esophageal rupture may contribute to the high possibility of primary repair and reduce severe mediastinal contamination and sepsis.^3^ If esophageal rupture is misdiagnosed, intraoperative transesophageal echocardiography may exacerbate the lesion and mediastinal contamination. On the other hand, the intervention timing and pattern also depend on the extent of emergency of the combined aortic injury. Fortunately, the occult injury to the esophagus was suspected on CT and finally confirmed with endoscopy in our case, which also changed the whole treatment strategy.

Traumatic AD may attract more attention and be given urgent treatment, including conservative management, endovascular repair, and open surgery.^4^ Conservative management may be appropriate for selected patients with hemodynamic stability. Endovascular treatment has gradually replaced traditional open surgery for type B AD.^5^ As for type A AD, open surgery to replace the involved ascending aorta or aortic arch is still preferred,^6^ although varied modified “smoke tube-and-window” techniques with cover stents have been tried.^7^ In general, immediate aortic repair may be needed, especially when the patient is unstable or has active bleeding from a high-grade aortic injury. Our patient was given conservative therapy for AD at the first stage because of hemodynamic stability and the low possibility of aortic lesion progression, and he accepted definite aortic repair successfully when his general condition was improved. In addition, limited aortic repair was applied in the case instead of more aggressive techniques, such as ascending aorta or arch replacement and aortic root repair or replacement.

Hence, strategies for definite treatment of aortic injury should be chosen on the basis of urgency and complexity of combined injuries as well as personalized aortic lesions. Delaying the repair and allowing time for the stress of the trauma to subside may have a better prognosis in the polytrauma AD patient with a low risk of aortic rupture or cardiac tamponade.
